# Cutaneous sporotrichosis after contact with a domestic cat

**DOI:** 10.1590/0037-8682-0074-2026

**Published:** 2026-06-15

**Authors:** Júlia de Souza Lopes, Ianara Castor Soares, Luana Ferreira Marques Cordeiro, Joziana Muniz de Paiva Barçante, Leonardo Lima Gorza

**Affiliations:** 1 Universidade Federal de Lavras, Departamento de Medicina, Lavras, MG, Brasil.; 2 Universidade Federal de Lavras, Programa de Pós-graduação em Ciências Veterinárias, Lavras, MG, Brasil.; 3 Universidade Federal de Lavras, Núcleo de Pesquisa Biomédica, Faculdade de Ciências da Saúde, Lavras, MG, Brasil.; 4 Universidade Federal de Lavras, Setor de Patologia Humana, Departamento de Medicina, Lavras, MG, Brasil.

**FIGURE 1:**
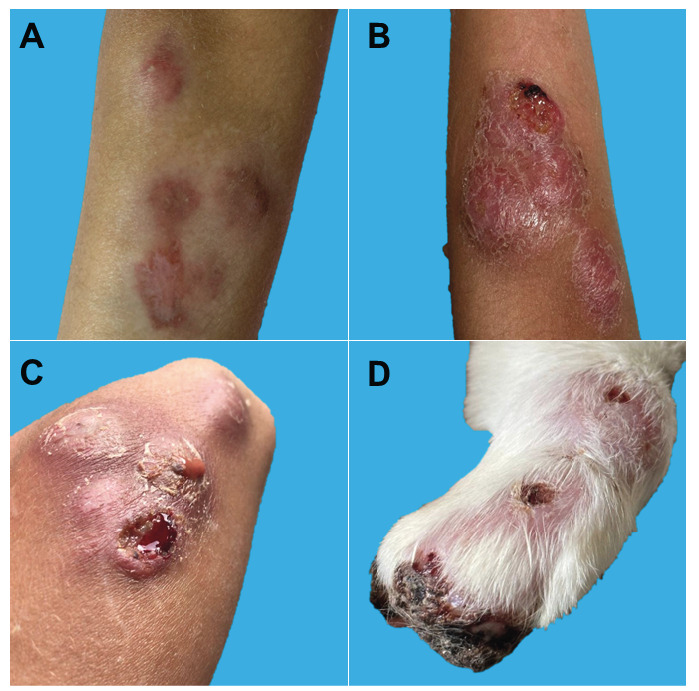
**(A):** Early erythematous nodular lesion before ulceration. **(B):** Ulcerated cutaneous nodules with irregular borders affecting the girl’s upper limb, and **(C):** the mother’s lower limb. **(D):** An ulcerative lesion was observed on the right thoracic limb of the domestic cat associated with these cases.

A 38-year-old woman and a 12-year-old girl were admitted to the health service center with erythematous, ulcerated cutaneous nodules. According to the patients, the lesions developed after contact with the family's domestic cat. Initially, a single painless erythematous nodule measuring approximately 2 cm in diameter was observed (Figure 1-A). Cutaneous nodules were noted on the neck, arms, and legs. These nodules had a multifocal distribution, irregular borders, and ulcerated centers (Figure 1-B and C). 

No lymphadenopathy was observed. Skin scrapings were used for cytological evaluation of the nodules, and the slides were stained with Romanowsky´s rapid stain. Microscopically, small organisms, 3 to 5 μm in diameter, elongated, or cigar-shaped to oval, consistent with *Sporothrix* spp., were identified. The domestic cat had a focal ulcer on the right thoracic limb, and a diagnosis of cutaneous sporotrichosis was also established by cytological analysis (Figure 1-D ). Treatment for both patients consisted of oral itraconazole (100 mg, once daily, initially for 45 days), oral saturated solution of potassium iodide, and a combination of amoxicillin and clavulanate (20 mg/kg, twice daily, for 7 days). Complete remission of the cutaneous lesions was observed approximately 180 days after starting the treatment.

Cat-human contact is steadily increasing, underscoring the importance of domestic felines in the transmission of human sporotrichosis^1^ ^,^ ^2^. Human sporotrichosis is classified based on clinical features, and most affected patients are diagnosed with the lymphocutaneous form^3^ ^,^ ^4^. Diagnosis is established by integrating clinical history, lesion characteristics, and laboratory confirmation.
